# Toll-Like Receptor (*TLR2* and *TLR4*) Polymorphisms and Chronic Obstructive Pulmonary Disease

**DOI:** 10.1371/journal.pone.0043124

**Published:** 2012-08-28

**Authors:** Simona E. Budulac, H. Marike Boezen, Pieter S. Hiemstra, Therese S. Lapperre, Judith M. Vonk, Wim Timens, Dirkje S. Postma, the GLUCOLD study group

**Affiliations:** 1 Department of Epidemiology, University of Groningen, University Medical Center Groningen, Groningen, The Netherlands; 2 Department of Pulmonology, Leiden University Medical Center, Leiden, The Netherlands; 3 Department of Pathology, University of Groningen, University Medical Center Groningen, Groningen, The Netherlands; 4 Department of Pulmonology, University of Groningen, University Medical Center Groningen, Groningen, The Netherlands; 5 The GLUCOLD study group: Groningen Leiden Universities Corticosteroids in Obstructive Lung Disease; 6 Groningen Research Institute for Asthma and COPD (GRIAC), University of Groningen, University Medical Center Groningen, Groningen, The Netherlands; Statens Serum Institute, Denmark

## Abstract

Toll-like receptors (TLRs) participate in the defence against bacterial infections that are common in patients with Chronic Obstructive Pulmonary Disease (COPD). We studied all tagging SNPs in *TLR2* and *TLR4* and their associations with the level and change over time of both FEV_1_ and sputum inflammatory cells in moderate-to-severe COPD. Nine *TLR2* SNPs and 17 *TLR4* SNPs were genotyped in 110 COPD patients. Associations of SNPs with lung function and inflammatory cells in induced sputum were analyzed cross-sectionally with linear regression and longitudinally with linear mixed-effect models. Two SNPs in *TLR2* (rs1898830 and rs11938228) were associated with a lower level of FEV_1_ and accelerated decline of FEV_1_ and higher numbers of sputum inflammatory cells. None of the *TLR4* SNPs was associated with FEV_1_ level. Eleven out of 17 SNPs were associated with FEV_1_ decline, including rs12377632 and rs10759931, which were additionally associated with higher numbers of sputum inflammatory cells at baseline and with increase over time. This is the first longitudinal study showing that tagging SNPs in *TLR2* and *TLR4* are associated with the level and decline of lung function as well as with inflammatory cell numbers in induced sputum in COPD patients, suggesting a role in the severity and progression of COPD.

## Introduction

Chronic Obstructive Pulmonary Disease (COPD) is characterized by inflammation and tissue destruction which are partially maintained by the innate immune defence system [1]. The innate immune response in the airways involves the detection of pathogen- or damage-associated molecular patterns by recognition receptors such as Toll-like receptors (TLRs) on cell surfaces [2]. TLRs participate in the defence against viral and bacterial infections, and such infections contribute to disease progression of COPD. TLRs may thus have a role in COPD development and/or progression.

Especially TLR2 and TLR4 have been studied among the TLRs that recognize gram positive [3] and gram negative bacteria. TLR2 and TLR4 are highly expressed on neutrophils and monocytes/macrophages in COPD [4], [5]. The expression of TLR4, but not TLR2, is increased in neutrophils recovered from bronchoalveolar lavage fluid of smokers with COPD and acute respiratory failure [6] and from sputum of patients with stable COPD [5].

The potential impact of functional single nucleotide polymorphisms (SNPs) in the *TLR2* and *TLR4* genes on COPD has been previously investigated [7]–[10]. For instance Asp299Gly in the *TLR4* was shown to be associated with decreased lipopolysaccharide (LPS) signal transduction [11]. Moreover, the prevalence of Asp299Gly SNP (rs498670) in *TLR4* appeared to be lower in COPD patients than controls [8]. One study suggested that the same SNP, rs498670, might not have a major impact on COPD development, since no significant effects of this SNP on lung function were found [9]. Another study focusing on *TLR2* showed that Arg677Trp (no rs designation available) and Arg753Gln (rs5743708) are not associated with either the onset or the course of COPD [7]. So far, other SNPs in *TLR2* and *TLR4,* apart from the most extensively studied SNPs mentioned above, have not been studied in relation to COPD. Moreover, it is as yet unknown whether the SNPs in *TLR2* and *TLR4* have any effect on lung function decline or changes in the number of inflammatory cells involved in the innate immune response. Therefore, we investigated the association of all tagging SNPs in *TLR2* and *TLR4* with the level and decline of lung function and with the level and changes in inflammatory cells in induced sputum over time of subjects with established COPD (Groningen Leiden Universities and Corticosteroids in Obstructive Lung Disease; the GLUCOLD study).

## Methods

### Study population

We included 114 patients with stage II-III COPD (according to the GOLD criteria [12]) who participated in a two-center trial (the GLUCOLD study [13]; study protocol available at www.clinicaltrials.gov). Patient characteristics and methods have been described in detail previously [13]. The patients had irreversible airflow limitation and chronic respiratory symptoms [14] and had neither used a course of oral steroids during the previous 3 months, nor maintenance treatment with inhaled or oral steroids during the previous 6 months. They were current or ex-smokers with a smoking history of ≥10 packyears, aged between 45 and 75 years without a history of asthma. The study was approved by the medical ethics committees of the University Medical Centers of Leiden and Groningen. All patients gave their written informed consent.

### Clinical characteristics

Lung function and reversibility to salbutamol were measured as described previously [13]. Sputum induction and whole sample processing were performed as described previously [13] according to a validated technique [15] (details are presented in Materials S1). The patients were in clinically stable condition and had no symptoms or signs of respiratory tract infection for at least two weeks prior to the study and before each visit [13].

### Intervention and follow-up procedures

Patients with mild-moderate COPD were randomly assigned to receive either 1) fluticasone propionate, 500 µg twice daily, for the first 6 months followed by placebo, twice daily, for 24 months; 2) fluticasone, 500 µg twice daily for 30 months; 3) fluticasone, 500 µg twice daily and salmeterol, 50 µg twice daily, in a single inhaler for 30 months; 4) placebo, twice daily, for 30 months [16].

### Selection of the *TLR2* and *TLR4* tagging SNPs and genotyping

We selected the tagging SNPs in *TLR2* and *TLR4* according to HapMap CEU genotype data (release 24) with an r^2^ threshold of 0.8 [17] and Minor Allele Frequency (MAF)>1%, resulting in 9 and 17 tagging SNPs respectively. Genotyping was performed by K-Bioscience (UK) using their patent-protected competitive allele specific PCR system (KASPar). DNA was available from 110 out of 114 COPD patients [18].

### Statistics

Numbers of nonsquamous inflammatory cells in induced sputum were log transformed to achieve normal distribution. We used linear regression analyses to assess the associations of *TLR2* and *TLR4* SNPs with FEV_1_ level and with the number of inflammatory cells in induced sputum at baseline. We adjusted our analyses for age, gender, height, packyears and smoking status. Linear mixed-effect (LME) models were used to asses associations of the *TLR2* and *TLR4* SNPs with change in FEV_1_ and inflammatory cells in induced sputum from baseline. Analyses were adjusted for age, gender, height, smoking status, the corresponding initial baseline variable (e.g. for FEV_1_ decline adjusted for baseline FEV_1_), treatment, the period when there is a change in treatment and its interaction with treatment and the interaction of all variables with time. We performed LME models with a random intercept at the subject's level, assuming that data is missing at random.

To assess the associations of the *TLR2* and *TLR4* SNPs with the outcomes of the present study we used the general genetic model with heterozygote and homozygote variants coded separately as dummy variables and compared them with the homozygote wild type.

Analyses were performed using SPSS version 18.0 for Windows and values of p<0.05 (tested 2-sided) were considered statistically significant.

## Results

The clinical characteristics of COPD patients are presented in Table 1 and the numbers of inflammatory cells in induced sputum in Table 2.

**Table 1:** Clinical characteristics of COPD patients.

	COPD patients (n = 114)
Males, n (%)	99 (86.8)
Age (years)	61.6 (7.7)
Height (cm)	175.5 (7.8)
Packyears*	41.8 (31.2–54.7)
Current smoker, n (%)	72 (63.2)
FEV_1_ % pred.**	56 (10)
FEV_1_ (L)	1.8 (0.4)
FEV_1_/IVC (%)	49.5 (8.8)

Data are presented as mean (standard deviation) or

* median (25^th^–75^th^ percentile); FEV_1_ = Forced Expiratory Volume in one second; FEV_1_/IVC = FEV_1_/Inspiratory Vital Capacity;

** % pred. = percentage of predicted value.

**Table 2:** The number of non-squamous inflammatory cells in induced sputum.

Induced sputum	Absolute numbers (10^4^/ml)	Percentage (%)
Total cell count*	139.7 (77.9–311.3)	-
Neutrophils	101.6 (46.8–228.5)	72.8 (59.9–81.7)
Macrophages	31.1 (17.9–61.1)	22.1 (14.8–33.2)
Eosinophils	1.3 (0.4–4.5)	1.1 (0.3–2.2)
Lymphocytes	2.2 (1.1–6.8)	1.7 (1.2–2.3)
Epithelial cells	1.4 (0.6–3.4)	1.0 (0.3–2.3)

Data are presented as median (25^th^–75^th^ percentile).

* Total cell count refers to the number of non-squamous cells in induced sputum.

All SNPs in *TLR2* and *TLR4* were in Hardy Weinberg Equilibrium (p>0.05) and were not correlated with each other (r^2^<0.8). The prevalence of the SNPs in *TLR2* and *TLR4* is presented in Table S1 and Table S2.

### 1. SNPs in *Toll-like receptor 2* and FEV_1_ level at baseline and FEV_1_ decline

Individuals homozygote for rs1898830 and rs11938228 had a significantly lower FEV_1_ level at baseline compared with wild-type individuals [B (95%CI = −267 ml (−522.8–−13.2) and −240.2 ml (−450.3–30.1)] (Table 3). Individuals heterozygote for rs7656411 and rs4696480 had a significantly higher FEV_1_ level at baseline compared with wild-type individuals [185 ml (55.1–316.2) and 170.2 ml (22.6–317.8) respectively] (Table 3).

**Table 3:** *TLR2* SNPs and FEV_1_ level at baseline and FEV_1_ decline.

SNP		FEV_1_ level (ml)	p	FEV_1_ decline (ml/yr)	p
		B (95%CI)		E (95%CI)	
rs1898830	a	−95.5 (−222.8–31.8)	0.140	−2.4 (−4.4–−0.4)	**0.021**
	b	−267.9 (−522.8–−13.2)	**0.039**	5.2 (1.4–9.0)	**0.008**
rs3804099	a	140.8 (−8.3–289.9)	0.064	1.5 (−0.9–3.8)	0.219
	b	118.6 (−66.9–304.1)	0.208	3.1 (0.2–5.9)	**0.036**
rs3804100	a	116.3 (−57.9–290.5)	0.188	2.5 (−0.2–5.2)	0.073
rs1816702	a	45.8 (−102.7–194.2)	0.542	0.1 (−2.3–2.4)	0.977
	b	169.9 (−164.5–504.4)	0.316	−8.2 (−12.9–−3.4)	**0.001**
rs11938228	a	−73.0 (−202.4–56.5)	0.266	−2.2 (−4.2–−0.1)	**0.042**
	b	−240.2 (−450.3–−30.1)	**0.025**	3.1 (−0.2–6.3)	0.065
rs7656411	a	185.6 (55.1–316.2)	**0.006**	1.9 (−0.2–4.0)	0.080
	b	−37.6 (−300.8–225.6)	0.778	8.6 (4.7–12.4)	**1.3×10^−5^**
rs5743704	a	114.4 (−123.0–351.8)	0.341	−2.8 (−6.5–0.9)	0.130
rs5743708	a	−72.1 (−268.8–124.7)	0.469	−0.1 (−3.1–2.8)	0.931
rs4696480	a	170.2 (22.6–317.8)	**0.024**	−1.7 (−4.1–0.6)	0.143
	b	108.2 (−62.1–278.5)	0.210	1.1 (−1.6–3.8)	0.413

FEV_1_ level adjusted for age, gender, height, pack-year, current smoking; FEV_1_ decline adjusted for FEV_1_ baseline, age, gender, height, current smoking, treatment, the period when there is a change in treatment and its interaction with treatment and their interaction with time; a = heterozygotes vs. wild-type; b = homozygote variant vs. wild-type, p = p-value.

Individuals heterozygote for rs1898830 and rs11938228 had a significantly accelerated FEV_1_ decline compared with wild-type individuals [−2.4 ml/yr (−4.4–−0.4) and −2.2 ml/yr (−4.2–−0.1) respectively]. Individuals homozygote for rs1898830, rs3804099 and rs7656411 had significantly less FEV_1_ decline compared with wild-type individuals [5.2 ml/yr (1.4–9.0), 3.1 ml/yr (0.2–5.6) and 8.6 ml/yr (4.7–12.4) respectively] (Table 3). Other SNPs were not significantly associated.

### 2. SNPs in *Toll-like receptor 2* and inflammatory cells in induced sputum: baseline level and changes from baseline

**Figure 1:**
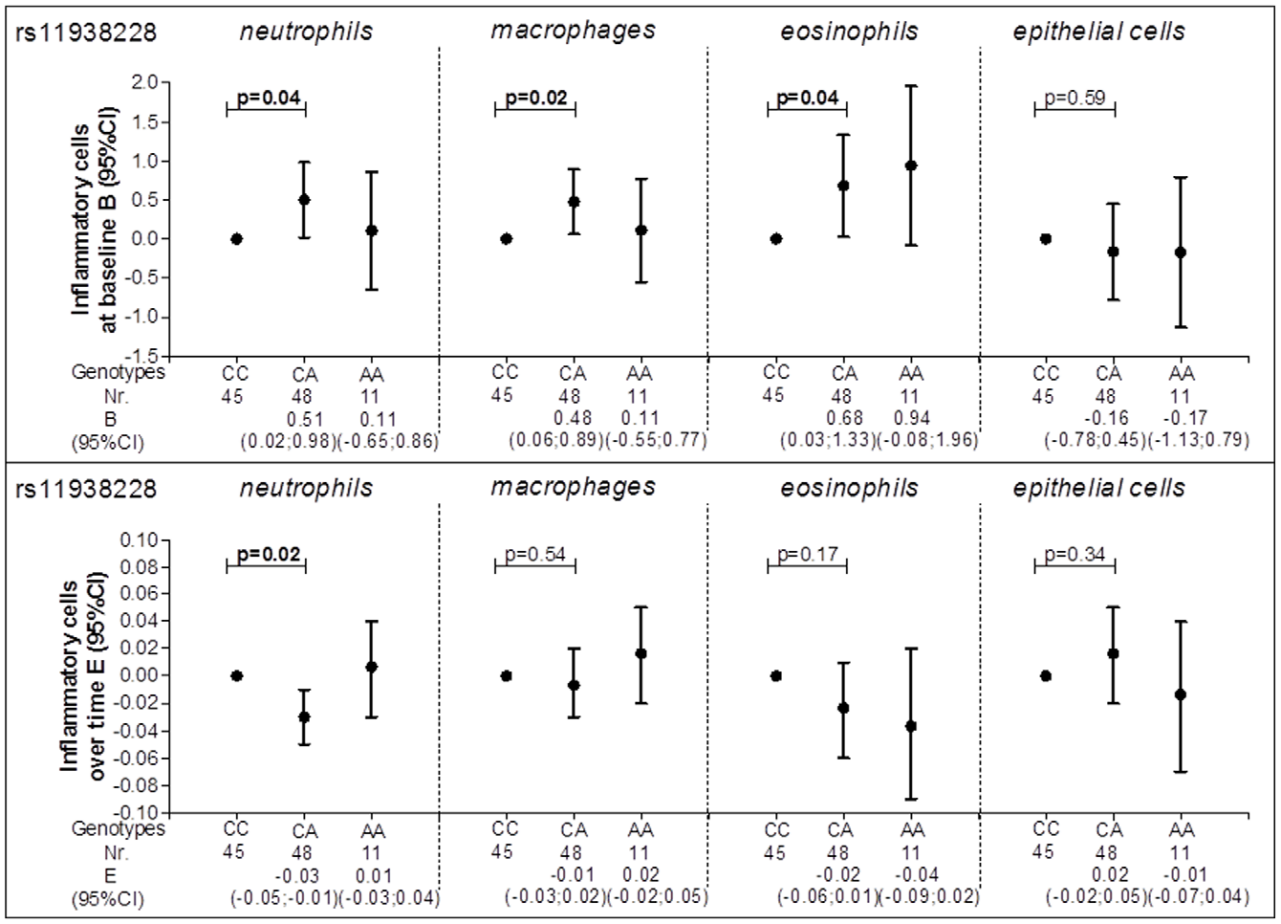
rs11938228 and inflammatory cells in induced sputum. Circles represent the regression coefficient (estimate) and vertical bars the 95% confidence interval (CI); Nr. = number of subjects; Wild type was set as the reference category (CC); At baseline analyses are adjusted for age, gender, height, packyears and smoking status; Over time analyses are adjusted for age, gender, height, smoking status, the corresponding initial baseline variable, treatment, the period when there is a change in treatment and its interaction with treatment and the interaction of all variables with time.

At baseline, individuals heterozygote for rs11938228 had a significantly higher number of sputum neutrophils, macrophages and eosinophils compared with wild-type individuals [0.51 (0.02–0.98), 0.48 (0.06–0.89) and 0.68 (0.03–1.33) respectively] (Figure 1). Individuals heterozygote for the same SNP had a significant decrease in sputum neutrophil numbers over time compared with the wild-type individuals [−0.03 (−0.05–−0.01)] (Figure 1).

Individuals heterozygote for rs3804099 had a significant decrease in neutrophil and macrophage numbers over time compared with wild-type individuals [−0.03 (−0.05–−0.01) and −0.04 (−0.06–−0.01) respectively] (Table S3 and Table S4).

The detailed results of the *TLR2* SNPs and inflammatory cells in induced sputum are presented in the data supplement (Table S3, Table S4, Table S5 and Table S6).

### 3. SNPs in *Toll-like receptor 4* and FEV_1_ level at baseline and FEV_1_ decline

None of the TLR4 SNPs was significantly associated with FEV_1_ level at baseline.

Individuals homozygote for rs2737190, rs1927911, rs7846989, rs7037117 and rs10983755 had a significantly accelerated FEV_1_ decline compared with wild-type individuals [−5.0 ml/yr (−8.7–−1.3), −5.4 ml/yr (−9.6–−1.1), −11.0 ml/yr (−20.2–−1.8), −9.1 ml/yr (−15.1–−3.2) and −10.7 ml/yr (−19.9–−1.5) respectively] (Table 4).

**Table 4:** *TLR4* SNPs and FEV_1_ level at baseline and FEV_1_ decline.

SNP		FEV_1_ level (ml)	p	FEV_1_ decline (ml/yr)	p
		B (95%CI)		E (95%CI)	
rs2770150	a	33.3 (−97.8–164.3)	0.615	1.7 (−0.3–3.7)	0.097
	b	193.2 (−86.3–472.6)	0.173	−1.6 (−7.1–4.1)	0.587
rs2737190	a	11.3 (−123.1–145.5)	0.869	0.8 (−1.3–2.7)	0.469
	b	84.6 (−167.3–336.5)	0.507	−5.0 (−8.7–−1.3)	**0.008**
rs10759932	a	−36.9 (−195.6–121.8)	0.646	−2.0 (−4.3–0.3)	0.084
	b	−19.8 (−503.8–463.6)	0.935	−4.6 (−10.4–1.3)	0.123
rs1927911	a	56.5 (−77.5–190.4)	0.405	−0.2 (−2.2–1.9)	0.872
	b	136.6 (−144.8–417.9)	0.338	−5.4 (−9.6–−1.1)	**0.014**
rs4986790	a	−71.9 (−277.5–133.8)	0.490	0.8 (−2.4–3.9)	0.633
rs11536889	a	−7.7 (−147.2–131.9)	0.914	0.8 (−1.4–2.9)	0.470
	b	−230.8 (−613.6–151.9)	0.234	−1.2 (−6.6–4.3)	0.682
rs7856729	a	144.8 (−8.3–297.9)	0.063	1.5 (−1.0–3.9)	0.244
	b	−6.3 (−467.0–454.5)	0.979	−7.3 (−13.8–−0.8)	**0.028**
rs7846989	a	−168.6 (−348.2–11.0)	0.065	−0.9 (−3.5–1.7)	0.489
	b	−60.9 (−732.5–610.7)	0.858	−11.0 (−20.2–−1.8)	**0.020**
rs7037117	a	46.8 (−245.6–339.1)	0.743	−5.4 (−9.2–−1.7)	**0.005**
	b	246.7 (−301.1–794.4)	0.360	−9.1 (−15.1–−3.2)	**0.003**
rs10983755	a	−118.2 (−412.6–176.3)	0.428	−3.3 (−7.8–1.2)	0.151
	b	−43.9 (−712.5–624.7)	0.897	−10.7 (−19.9–−1.5)	**0.023**
rs12377632	a	−7.5 (−150.1–135.1)	0.917	2.4 (0.1–4.7)	**0.040**
	b	−110.4 (−309.1–88.3)	0.273	0.4 (−2.7–3.5)	0.793
rs11536857	a	−92.9 (−332.3–146.4)	0.443	−0.6 (−4.4–3.2)	0.753
	b	−180.2 (−484.4–123.9)	0.243	0.2 (−4.1–4.5)	0.923
rs11536869	a	−106.1 (−491.7–279.5)	0.586	6.4 (1.1–11.7)	**0.019**
rs913930	a	−15.6 (−157.6–126.3)	0.828	1.4 (−0.7–3.5)	0.204
	b	62.9 (−184.3–310.1)	0.615	1.8 (−2.4–5.9)	0.399
rs11536897	a	110.2 (−115.5–335.9)	0.335	7.5 (3.8–11.3)	**9.1×10^−5^**
rs10759931	a	−3.3 (−152.9–146.4)	0.966	2.6 (0.2–4.9)	**0.033**
	b	−94.1 (−292.2–104.0)	0.348	−0.9 (−3.9–2.1)	0.534
rs11536878	a	143.9 (−28.4–316.1)	0.101	3.5 (0.5–6.4)	**0.021**
	b	317.1 (−52.2–686.4)	0.092	−5.7 (−14.8–3.4)	0.217

FEV_1_ level adjusted for age, gender, height, pack-year, current smoking; FEV_1_ decline adjusted for FEV_1_ baseline, age, gender, height, current smoking, treatment, the period when there is a change in treatment and its interaction with treatment and their interaction with time; a = heterozygotes vs. wild-type; b = homozygote variant vs. wild-type, p = p-value.

Individuals heterozygote for rs12377632, rs11536869, rs11536897, rs10759931 and rs11536878 had significantly less FEV_1_ decline compared with wild-type individuals [2.4 ml/yr (0.1–4.7), 6.4 ml/yr (1.1–11.7), 7.5 ml/yr (3.8–11.3), 2.6 ml/yr (0.2–4.9) and 3.5 ml/yr (0.5–6.4) respectively] (Table 4).

### 4. SNPs in *Toll-like receptor 4* and inflammatory cells in induced sputum: baseline level and changes from baseline

**Figure 2:**
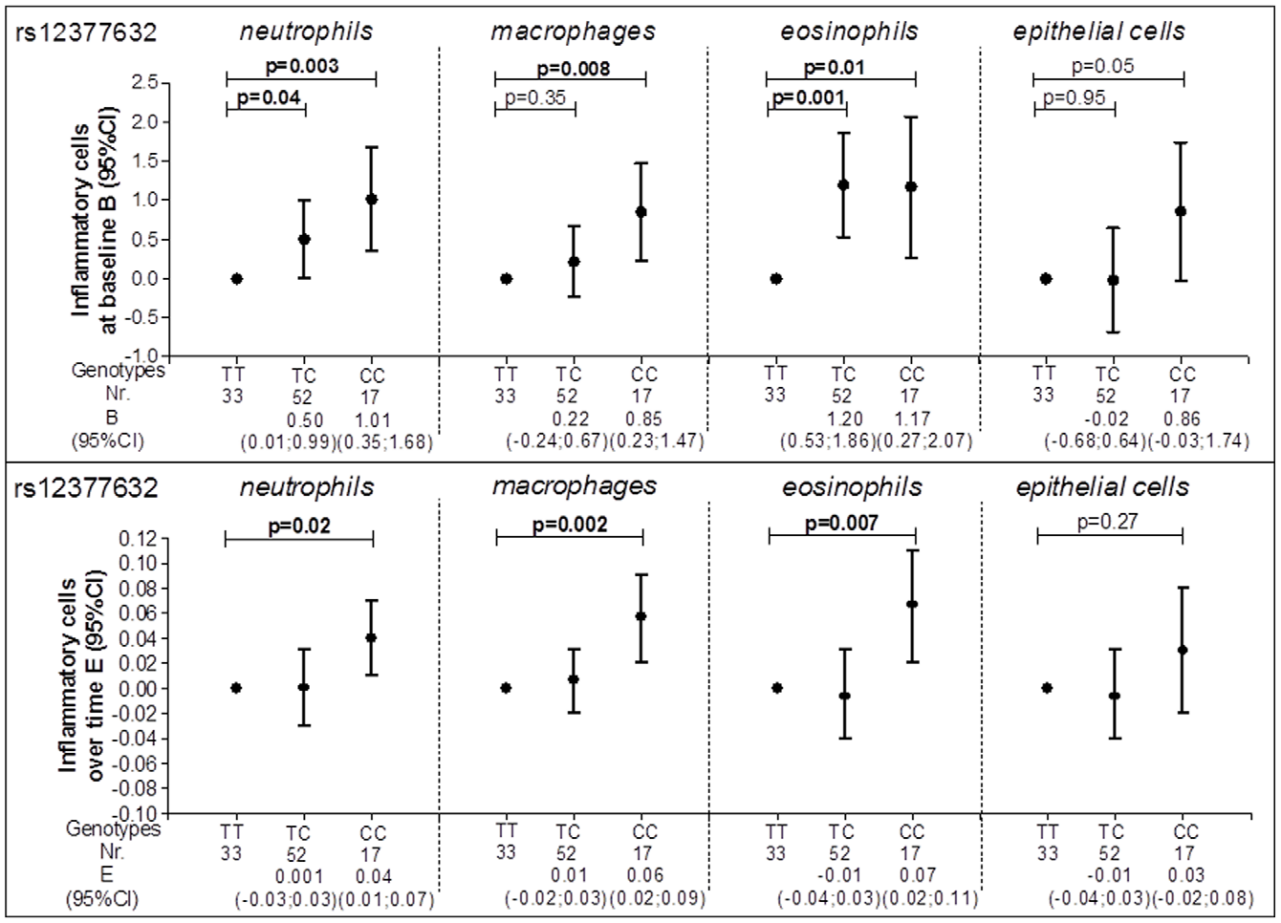
rs12377632 and inflammatory cells in induced sputum. Circles represent the regression coefficient (estimate) and vertical bars the 95% confidence interval (CI); Nr. = number of subjects; Wild type was set as the reference category (TT); At baseline analyses are adjusted for age, gender, height, packyears and smoking status; Over time analyses are adjusted for age, gender, height, smoking status, the corresponding initial baseline variable, treatment, the period when there is a change in treatment and its interaction with treatment and the interaction of all variables with time.

At baseline individuals heterozygote for rs12377632 had a higher number of sputum neutrophils and eosinophils compared with wild-type individuals [0.50 (0.01–0.99) and 1.20 (0.53–1.86) respectively] (Figure 2). Individuals homozygote for the same SNP (rs12377632) had a significantly higher number of sputum neutrophils, macrophages and eosinophils at baseline compared with wild-type individuals [1.01 (0.35–1.68), 0.85 (0.23–1.47) and 1.17 (0.27–2.07) respectively] and also a significant increase in numbers over time [0.04 (0.01–0.07), 0.06 (0.02–0.09) and 0.07 (0.02–0.11) respectively] (Figure 2).

**Figure 3:**
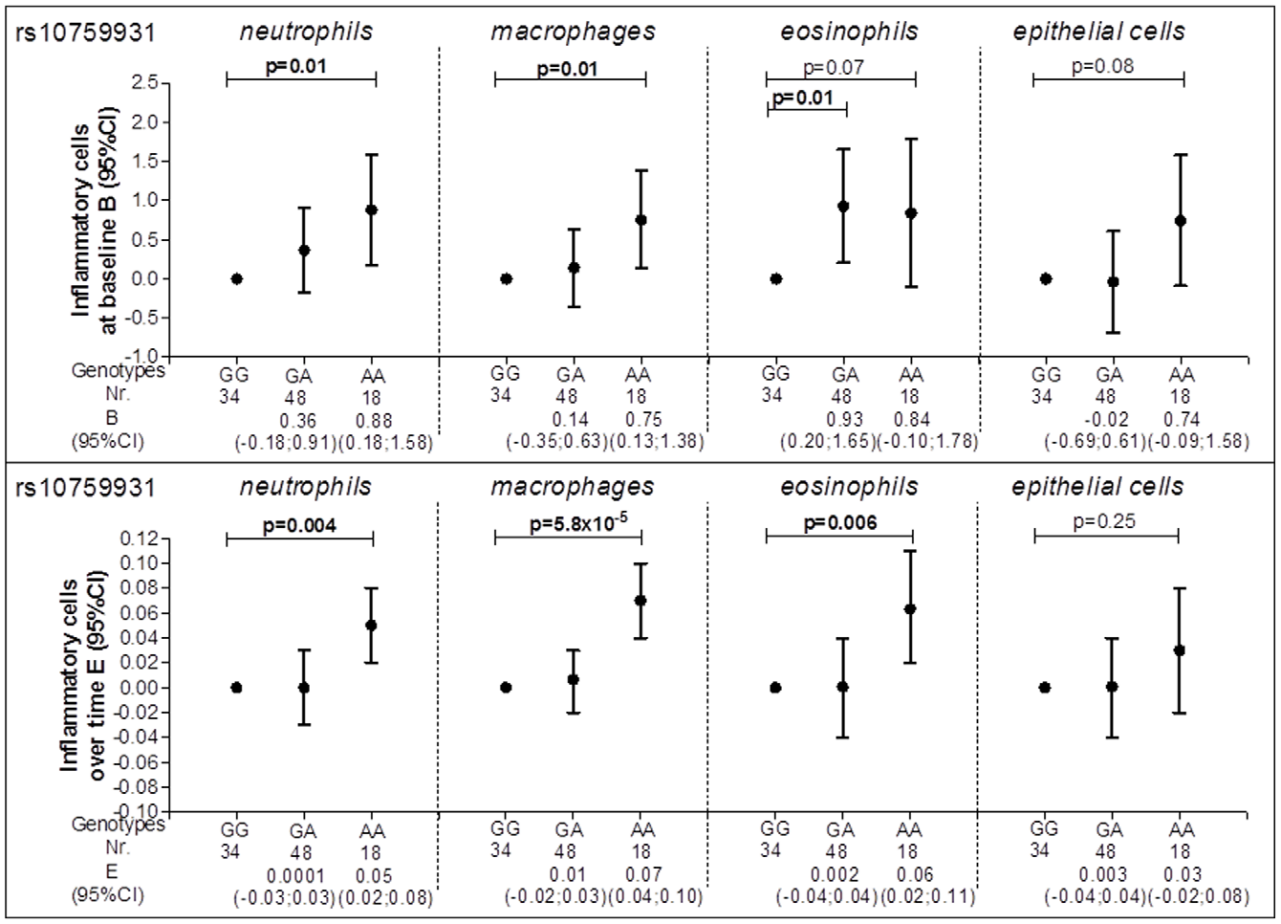
rs10759931 and inflammatory cells in induced sputum. Circles represent the regression coefficient (estimate) and vertical bars the 95% confidence interval (CI); Nr. = number of subjects; Wild type was set as the reference category (GG); At baseline analyses are adjusted for age, gender, height, packyears and smoking status; Over time analyses are adjusted for age, gender, height, smoking status, the corresponding initial baseline variable, treatment, the period when there is a change in treatment and its interaction with treatment and the interaction of all variables with time.

Individuals homozygote for rs10759931 had significantly higher sputum neutrophil and macrophage numbers at baseline compared with wild-type individuals [0.88 (0.18–1.58) and 0.75 (0.13–1.38) respectively] as well as a higher increase in numbers over time [0.05 (0.02–0.08) and 0.07 (0.04–0.10) respectively] (Figure 3). Individuals heterozygote for the same SNP (rs10759931) had a significantly higher number of eosinophils at baseline and individuals homozygote for the same SNP a significantly higher increase number of eosinophils over time compared with wild-type individuals [0.93 (0.20–1.65) and 0.06 (0.02–0.11) respectively] (Figure 3).

Individuals carrying rs913930 had significantly lower numbers of macrophages and eosinophils at baseline compared with wild-type individuals [−0.5 (−0.9–−0.1) and −1.7 (−2.8–−0.6) in heterozygote and homozygote variants respectively] and a decrease in numbers over time [−0.03 (−0.05–−0.01) and −0.07 (−0.13–−0.01) respectively] (Table S8 and Table S9).

Individuals heterozygote for rs2770150 had a significantly lower number of neutrophils at baseline compared with wild-type individuals [−0.48 (−0.94–−0.03)] and a larger fall in numbers of neutrophils, macrophages, eosinophils and epithelial cells over time [−0.03 (−0.05; −0.01), −0.02 (−0.04–−0.001), −0.05 (−0.08–−0.02) and −0.04 (−0.07–−0.002) respectively] (Table S7, Table S8, Table S9 and Table S10).

## Discussion

This is the first study investigating comprehensively the genetic contribution of Toll-like receptors to both the severity and the progression of COPD with respect to lung function and inflammation. We investigated 26 tagging SNPs in *TLR2* and *TLR4* and showed consistent associations with the level and decline of FEV_1_ as well as with inflammatory cell numbers in induced sputum at baseline and their changes over time.

We found that many SNPs in *TLR2* and *TLR4* were of relevance: 4 out of 9 SNPs in *TLR2* were significantly associated with the level of lung function, 2 with a lower level of FEV_1_ (rs1898830 and rs11938228) and 2 with a higher level of FEV_1_ (rs7656411 and rs4696480). The SNPs rs1898830, rs3804099 and rs7656411 were significantly associated with less FEV_1_ decline and the intronic SNPs rs1898830, rs1816702 and rs11938228 with accelerated FEV_1_ decline. The SNP rs11938228 was also associated with an increase in numbers of neutrophils, macrophages and eosinophils in sputum at baseline and with a decrease in numbers of neutrophils over time from baseline, suggesting that these features may either be associated or be influenced by one underlying mechanism. Rs3804099 was significantly associated with a decrease in neutrophils and macrophages over time.

None of the SNPs in *TLR4* was associated with the level of FEV_1_, whereas 11 out of 17 SNPs in *TLR4* were significantly associated with accelerated (n = 7) or reduced decline of FEV_1_ (n = 4). Rs12377632 and rs10759931 in *TLR4* were consistently associated with higher numbers of inflammatory cells in induced sputum at baseline and an increase in numbers of inflammatory cells in induced sputum over time from baseline. The other SNPs were associated with either the level or the changes in sputum inflammatory cell numbers from baseline.

Toll-like receptors form a component of the innate immune response which is the first line of defence against invading microorganisms. In humans, 10 functional TLRs have been described [19]. Each TLR expressed in the cellular membrane recognizes molecules such as the lipoproteins of gram-positive bacteria (TLR2) and LPS of gram-negative bacteria (TLR4) [19]. TLRs have been broadly studied in the perspective of microbial and viral infections, inflammation and immune cells [20], but not extensively in COPD. Findings on TLR2 expression on alveolar macrophages, sputum neutrophils and blood monocytes [5], [21], [22] do suggest a role for this receptor in inflammation that is a characteristic of COPD.

Even less is known on SNPs in *TLR2* and *TLR4* and COPD. Two SNPs in *TLR2* (rs1898830 and rs4696480) are associated with a lower respectively higher level of FEV_1_ in the current study and rs1898830 additionally with the decline of FEV_1_, suggesting that this SNP might be involved in the progression of the disease as well. This is compatible with observations in asthma where the same SNPs have been shown to modify the effect of PM_2.5_ exposure on the prevalence of asthma from birth up to 8 years of age [23]. Rs1898830, located in intron 1, was previously associated with TLR2-mediated cellular activation [24]. The authors suggested that this effect might be caused by the effect of the rs13150331 on transcriptional activities of the *TLR2* gene promoter, a SNP in the 5′-flanking region that is in strong LD with rs1898830 [24]. The observed associations in the current study may also be due to the effects of other SNPs in *TLR2* in LD with the intronic SNPs rs1898830 and rs4696480. However, there is accumulating evidence that mutations in the splice, donor and acceptor sites or enhancer, intron and promoter elements may all be important in genetic expression and regulation [25]. Therefore, functional assays are of interest to elucidate the molecular mechanisms underlying these associations.

We confirmed previous observations that there is no significant association of rs5743708 (Arg753Gln), a SNP shown to affect transmembrane signalling of *TLR2* [26], with progression of COPD [7]. Furthermore there was no association with numbers of inflammatory cells in induced sputum, suggesting that this specific SNP may have no impact on COPD.

Interestingly, rs11938228, an intronic *TLR2* SNP that has not been previously studied, was associated with a lower level of FEV_1_ and increased numbers of inflammatory cells in induced sputum at baseline, suggesting that this SNP negatively affects the severity of COPD.

A reduced *TLR4* gene expression has been found in nasal epithelium of smokers and severe COPD patients [27]. Other studies showed that expression of *TLR4* mRNA is inhibited by LPS in a mouse macrophage cell line [28] and stimulated in human neutrophils and monocytes [29]. This apparent discrepancy may reflect the differences in cell type and/or differentiation stages [20]. These findings are of interest given our observation that different SNPs in *TLR4* are associated either with the number of neutrophils or the number of macrophages (Table S8 and Table S9). Two SNPs in *TLR4* namely rs12377632 and rs10759931 were consistently associated with lung function and inflammation. However, the results from baseline and longitudinal analyses for heterozygotes and homozygotes separately require careful interpretation. For instance both heterozygotes and homozygotes for rs12377632 had a non-significantly lower level of lung function, but a significantly higher number of inflammatory cells in induced sputum, suggesting that this particular SNP may be involved in inflammatory processes representing the first line of defence as reflected in sputum. Additionally, heterozygote individuals for rs12377632 had less lung function decline, while homozygote individuals for the same SNP had a significantly higher number of inflammatory cells over time. This may signify that disease modification can be achieved for particular genotypes in COPD and that changes in induced sputum may represent an activation of the first line of defence yet this is not interrelated with changes in lung function.

Rs10759931 in *TLR4* may have functional consequences on TLR4 expression or signalling activity given its location in the promoter region. This may influence exclusively innate immunity and inflammation, which in turn may affect COPD severity and progression. Therefore, its association with less accelerated FEV_1_ decline and increase in numbers of macrophages over time is intriguing since one generally would anticipate that an increase in inflammatory burden associates with accelerated lung function decline. It is thus difficult to reconcile these two observations. This may be due to other genetic effects interacting with this particular SNP and/or differential effects of this SNP on many other underlying mechanisms of changes in lung function, like extracellular matrix turnover or effects on oxidative stress responses. Moreover, gene expression profiling combined with genetics should elucidate whether this SNP indeed is an eQTL (expression quantitative trait locus). Since rs10759931 could be a GATA2 binding site [23], future studies should unravel how the studied SNPs functionally contribute to COPD severity and progression.

Only 2 SNPs in *TLR4* have been previously investigated with respect to COPD; Asp299Gly (rs498670) and Thr399Ile (rs498671) [8]–[10]. Rs498670 appeared not to be present among patients with COPD who had never smoked [8] and there were no homozygote variants for rs498670 in smokers from the general population (>10 packyears and >40 years of age) [8], comparable to our study. We also confirm the previous findings that the presence of the *TLR4* rs498670 did not have any significant impact on lung function level [8] and extend this observation by showing that it was also not significantly associated with FEV_1_ decline. This indicates that rs498670 has no impact on the severity of COPD at a population level as well as in patients with established COPD.

We here show that rs2770150 was consistently associated with lower numbers of inflammatory cells in induced sputum in COPD although the effect size was small. This promoter SNP might thus positively influence inflammation in COPD patients. Since signalling through TLR2 and TLR4 by hyaluronan may be important in the maintenance of epithelial integrity in the lung after inflammatory insults and in repair [30], it could be that *TLR2* and *TLR4* SNPs are also exhibiting effects in the inflammatory processes in COPD in order to down regulate detrimental signals. More studies are clearly needed to validate these findings and to understand the mechanism by which the *TLR2* and *TLR4* polymorphisms affect the pathological role of TLRs in the signalling pathways involved in COPD, in particular taking into account the effects on level of expression of these receptors on different cell types. For instance there is an increased expression of TLR4 and TLR9 on lung CD8^+^ T cells [31].

It has been speculated that TLRs could delay FEV_1_ decline and thus serve as a therapy target for COPD patients [31], [32]. Of reference to our study, COPD patients have reduced TLR4 expression in epithelial cells and corticosteroids dose dependently reduced TLR4 mRNA in an epithelial cell line [27] and increased TLR2 expression [33]. Our patients used inhaled corticosteroids in the randomized GLUCOLD study [16]. Therefore, we adjusted for the period with a change in treatment and its interaction with treatment to avoid any interference with the treatment response. Due to the relatively low number of participants in the study and hence low power, a formal study on gene-treatment interaction was not feasible. However, even with the low numbers of individuals, we were able to find consistent associations of SNPs in *TLR2* and *TLR4* with level and decline of lung function and number of inflammatory cells in induced sputum. Although effects on lung function decline are small, they are consistent for different SNPs. Given the consistency of our results, it is of importance that future studies with a larger sample size of COPD patients confirm the clinical significance of our findings.

In the current dataset we did not apply a multiple testing correction (i.e. Bonferroni) given the clustering of outcome variables, which might occur jointly at high or low levels account (e.g. a Pearson's correlation coefficient r = 0.79 for macrophages and lymphocytes in induced sputum) or their definition as each other's ratios [16].

In summary, previous studies focused on functional SNPs only and studied COPD development exclusively, whereas our study is the first with longitudinal data showing that tagging SNPs in the *TLR2* and *TLR4* genes are associated with the level and decline of lung function as well as with (changes in) numbers of inflammatory cells in induced sputum. These different associations provide insights for future investigations on how these polymorphisms may produce different signatures of genes' activation and how they could eventually contribute to pharmacogenetics in COPD management, which will result in more accurate and targeted therapy.

## Supporting Information

Materials S1 **Supplementary methods.**
(DOC)

Table S1 **Prevalence of the** ***TLR2*** **SNPs.** N = number.
(DOC)

Table S2 **Prevalence of the** ***TLR4*** **SNPs.** N = number.
(DOC)

Table S3 ***TLR2*** **SNPs and neutrophils in induced sputum.** Baseline analysis are adjusted for age, gender, pack-year, current smoking; Change analysis are adjusted for neutrophils at baseline, age at baseline, gender, current smoking at baseline, treatment, the period when there is a change in treatment and its interaction with treatment and their interaction with time; a = heterozygotes vs. wild-type; b = homozygote variant vs. wild-type.
(DOC)

Table S4 ***TLR2*** **SNPs and macrophages in induced sputum.** Baseline analysis are adjusted for age, gender, pack-year, current smoking; Change analysis are adjusted for macrophages at baseline, age at baseline, gender, current smoking at baseline, treatment, the period when there is a change in treatment and its interaction with treatment and their interaction with time; a = heterozygotes vs. wild-type; b = homozygote variant vs. wild-type.
(DOC)

Table S5 ***TLR2*** **SNPs and eosinophils in induced sputum.** Baseline analysis are adjusted for age, gender, pack-year, current smoking; Change analysis are adjusted for eosinophils at baseline, age at baseline, gender, current smoking at baseline, treatment, the period when there is a change in treatment and its interaction with treatment and their interaction with time; a = heterozygotes vs. wild-type; b = homozygote variant vs. wild-type.
(DOC)

Table S6 ***TLR2*** **SNPs and epithelial cells in induced sputum.** Baseline analysis are adjusted for age, gender, pack-year, current smoking; Change analysis are adjusted for epithelial cells at baseline, age at baseline, gender, current smoking at baseline, treatment, the period when there is a change in treatment and its interaction with treatment and their interaction with time; a = heterozygotes vs. wild-type; b = homozygote variant vs. wild-type.
(DOC)

Table S7 ***TLR4*** **SNPs and neutrophils in induced sputum.** Baseline analysis are adjusted for age, gender, pack-year, current smoking; Change analysis are adjusted for neutrophils at baseline, age at baseline, gender, current smoking at baseline, treatment, the period when there is a change in treatment and its interaction with treatment and their interaction with time; a = heterozygotes vs. wild-type; b = homozygote variant vs. wild-type.
(DOC)

Table S8 ***TLR4*** **SNPs and macrophages in induced sputum.** Baseline analysis are adjusted for age, gender, pack-year, current smoking; Change analysis are adjusted for macrophages at baseline, age at baseline, gender, current smoking at baseline, treatment, the period when there is a change in treatment and its interaction with treatment and their interaction with time; a = heterozygotes vs. wild-type; b = homozygote variant vs. wild-type.
(DOC)

Table S9 ***TLR4*** **SNPs and eosinophils in induced sputum.** Baseline analysis are adjusted for age, gender, pack-year, current smoking; Change analysis are adjusted for eosinophils at baseline, age at baseline, gender, current smoking at baseline, treatment, the period when there is a change in treatment and its interaction with treatment and their interaction with time; a = heterozygotes vs. wild-type; b = homozygote variant vs. wild-type.
(DOC)

Table S10 ***TLR4*** **SNPs and epithelial cells in induced sputum.** Baseline analysis are adjusted for age, gender, pack-year, current smoking; Change analysis are adjusted for epithelial cells at baseline, age at baseline, gender, current smoking at baseline, treatment, the period when there is a change in treatment and its interaction with treatment and their interaction with time; a = heterozygotes vs. wild-type;b = homozygote variant vs. wild-type.
(DOC)
