# Supplementary material for: Toll-Like Receptor (TLR2 and TLR4) Polymorphisms and Chronic Obstructive Pulmonary Disease
Source: PLoS One. 2012 Aug 28;7(8):e43124. doi: 10.1371/journal.pone.0043124 (PMC3429472; doi:10.1371/journal.pone.0043124)
Supplement: Materials S1 — Supplementary methods. (DOC) [file pone.0043124.s001.doc]

**SUPPLEMENTARY MATERIAL**

***Toll-Like Receptors* (*TLR2* and *TLR4*) Polymorphisms and Chronic Obstructive Pulmonary Disease**

Simona E Budulac^1, 6^, **H Marike** Boezen^1, 6^, **Pieter S Hiemstra^2^, Therese S Lapperre^2^,** Judith M Vonk^1, 6^, Wim Timens^3, 6^ **and** Dirkje S Postma^4, 6, *^ and the GLUCOLD study group^5^

^1^Department of Epidemiology, University Medical Center Groningen, University of Groningen, Groningen, The Netherlands

^2^Department of Pulmonology, Leiden University Medical Center, Leiden, The Netherlands

^3^Department of Pathology, University Medical Center Groningen, University of Groningen, Groningen, The Netherlands

^4^Department of Pulmonology University Medical Center Groningen, University of Groningen, Groningen, The Netherlands

^5^The GLUCOLD study group: Groningen Leiden Universities Corticosteroids in Obstructive Lung Disease

^6^Groningen Research Institute for Asthma and COPD (GRIAC), University Medical Center Groningen, University of Groningen, Groningen, The Netherlands

**^*^Corresponding author:** SE Budulac, Department of Epidemiology, University Medical Center Groningen, PO Box 30.001, Hanzeplein 1, 9713 GZ Groningen, The Netherlands; e-mail: s.budulac@umcg.nl; Telephone: +31 50 361 1688; Fax: +31 50 3614493

**Supplementary methods**

**Clinical characteristics**

Sputum induction and processing were performed as described previously [1] according to a validated technique [2]. After inhaling 200 μg salbutamol, patients inhaled hypertonic sodium chloride aerosols (4.5% weight/volume) during three periods of 5 min. Differential cell counts were expressed as a percentage of nucleated cells, excluding squamous cells. A sputum sample was considered adequate when the percentage of squamous cells was <80%.

References

1. Lapperre TS, Snoeck-Stroband JB, Gosman MM, Stolk J, Sont JK, Jansen DF, Kerstjens HA, Postma DS, Sterk PJ. Dissociation of lung function and airway inflammation in chronic obstructive pulmonary disease. *Am J Respir Crit Care Med* 2004; 170: 499-504.

2. in 't Veen JC, de Gouw HW, Smits HH, Sont JK, Hiemstra PS, Sterk PJ, Bel EH. Repeatability of cellular and soluble markers of inflammation in induced sputum from patients with asthma
18. *Eur Respir J* 1996; 9: 2441-2447.
