# Supplementary material for: Toll-Like Receptor (TLR2 and TLR4) Polymorphisms and Chronic Obstructive Pulmonary Disease
Source: PLoS One. 2012 Aug 28;7(8):e43124. doi: 10.1371/journal.pone.0043124 (PMC3429472; doi:10.1371/journal.pone.0043124)
Supplement: Table S1 — Prevalence of the TLR2 SNPs. N = number. (DOC) [file pone.0043124.s002.doc]

**Table S1: Prevalence of the *TLR2* SNPs**

| **SNP** | **Genotypes** | **N (%)** | **Location** | **AA position** |
| --- | --- | --- | --- | --- |
| rs1898830 | AA | 46 (43.0) | Intron 1 | - |
|  | AG | 54 (50.5) |  |  |
|  | GG | 7 (6.5) |  |  |
| rs3804099 | TT | 27 (25.7) | Exon 3 | Asn199Asn |
|  | TC | 56 (53.3) |  |  |
|  | CC | 22 (21.0) |  |  |
| rs3804100 | TT | 93 (85.3) | Exon 3 | Ser450Ser |
|  | CT | 16 (14.7) |  |  |
|  | CC | 0 (0) |  |  |
| rs1816702 | CC | 79 (71.8) | Intron 2 | - |
|  | CT | 27 (24.5) |  |  |
|  | TT | 4 (3.6) |  |  |
| rs11938228 | CC | 45 (43.3) | Intron 2 | - |
|  | CA | 48 (46.2) |  |  |
|  | AA | 11 (10.6) |  |  |
| rs7656411 | TT | 64 (59.3) | 3’region | - |
|  | TG | 38 (35.2) |  |  |
|  | GG | 6 (5.6) |  |  |
| rs5743704 | CC | 98 (92.5) | Exon 3 | His631Pro |
|  | CA | 8 (7.5) |  |  |
|  | AA | 0 (0) |  |  |
| rs5743708 | GG | 95 (88.0) | Exon 3 | Gln753Arg |
|  | GA | 13 (12.0) |  |  |
|  | AA | 0 (0) |  |  |
| rs4696480 | TT | 29 (26.6) | Intron 1 | - |
|  | TA | 54 (49.5) |  |  |
|  | AA | 26 (23.9) |  |  |

N=number
