# Supplementary material for: Toll-Like Receptor (TLR2 and TLR4) Polymorphisms and Chronic Obstructive Pulmonary Disease
Source: PLoS One. 2012 Aug 28;7(8):e43124. doi: 10.1371/journal.pone.0043124 (PMC3429472; doi:10.1371/journal.pone.0043124)
Supplement: Table S2 — Prevalence of the TLR4 SNPs. N = number. (DOC) [file pone.0043124.s003.doc]

**Table S2: Prevalence of the *TLR4* SNPs**

| **SNP** | **Genotypes** | **N (%)** | **Location** | **AA position** |
| --- | --- | --- | --- | --- |
| rs2770150 | TT | 55 (50.9) | promotor | - |
|  | TC | 47 (43.5) |  |  |
|  | CC | 6 (5.6) |  |  |
| rs2737190 | AA | 50 (47.2) | promotor | - |
|  | AG | 47 (44.3) |  |  |
|  | GG | 9 (8.5) |  |  |
| rs10759932 | TT | 79 (75.2) | promotor | - |
|  | TC | 23 (21.9) |  |  |
|  | CC | 3 (2.9) |  |  |
| rs1927911 | CC | 61 (57.0) | Intron 1 | - |
|  | CT | 39 (36.4) |  |  |
|  | TT | 7 (6.5) |  |  |
| rs4986790 | AA | 93 (88.6) | Exon 3 | Asp299Gly |
|  | AG | 12 (11.4) |  |  |
|  | GG | 0 (0) |  |  |
| rs11536889 | GG | 75 (68.8) | 3’region | - |
|  | GC | 31 (28.4) |  |  |
|  | CC | 3 (2.8) |  |  |
| rs7856729 | GG | 80 (76.2) | 3’region | - |
|  | GT | 23 (21.9) |  |  |
|  | TT | 2 (1.9) |  |  |
| rs7846989 | TT | 89 (82.4) | 3’region | - |
|  | TC | 18 (16.7) |  |  |
|  | CC | 1 (0.9) |  |  |
| rs7037117 | AA | 19 (63.3) | 3’region | - |
|  | AG | 9 (30.0) |  |  |
|  | GG | 2 (6.7) |  |  |
| rs10983755 | GG | 101(93.5) | promotor | - |
|  | GA | 6 (5.6) |  |  |
|  | AA | 1 (0.9) |  |  |
| rs12377632 | TT | 33 (32.4) | Intron 2 | - |
|  | TC | 52 (51.0) |  |  |
|  | CC | 17 (16.7) |  |  |
| rs11536857 | CC | 96 (88.1) | promotor | - |
|  | CT | 8 (7.3) |  |  |
|  | TT | 5 (4.6) |  |  |
| rs11536869 | AA | 104 (97.2) | Intron 1 | - |
|  | AG | 3 (2.8) |  |  |
|  | GG | 0 (0) |  |  |
| rs913930 | TT | 45 (42.5) | 3’region | - |
|  | TC | 52 (49.1) |  |  |
|  | CC | 9 (8.5) |  |  |
| rs11536897 | GG | 99 (91.7) | 3’region | - |
|  | GA | 9 (8.3) |  |  |
|  | AA | 0 (0) |  |  |
| rs10759931 | GG | 34 (34.0) | promotor | - |
|  | GA | 48 (48.0) |  |  |
|  | AA | 18 (18.0) |  |  |
| rs11536878 | CC | 87 (82.1) | Intron 2 | - |
|  | CA | 16 (15.1) |  |  |
|  | AA | 3 (2.8) |  |  |

N=number
