# Supplementary material for: Toll-Like Receptor (TLR2 and TLR4) Polymorphisms and Chronic Obstructive Pulmonary Disease
Source: PLoS One. 2012 Aug 28;7(8):e43124. doi: 10.1371/journal.pone.0043124 (PMC3429472; doi:10.1371/journal.pone.0043124)
Supplement: Table S3 — TLR2 SNPs and neutrophils in induced sputum. Baseline analysis are adjusted for age, gender, pack-year, current smoking; Change analysis are adjusted for neutrophils at baseline, age at baseline, gender, current smoking at baseline, treatment, the period when there is a change in treatment and its interaction with treatment and their interaction with time; a = heterozygotes vs. wild-type; b = homozygote variant vs. wild-type. (DOC) [file pone.0043124.s004.doc]

**Table S3: *TLR2* SNPs and neutrophils in induced sputum**

|  |  | **(ln)neutrophils** |  | **(ln) neutrophils** |  |
| --- | --- | --- | --- | --- | --- |
| **SNP** |  | **baseline** | **p** | **change** | **p** |
|  |  | **B (95%CI)** |  | **E (95%CI)** |  |
| rs1898830 | a | 0.4 (-0.1 - 0.8) | 0.114 | -0.02 (-0.1 - 3.0x10^-5^) | *0.050* |
|  | b | -0.2 (-1.1 - 0.7) | 0.622 | -0.01 (-0.03 -0.05) | 0.746 |
| rs3804099 | a | 0.2 (-0.3 - 0.7) | 0.446 | -0.03 (-0.05 - -0.01) | **0.031** |
|  | b | -0.01 (-0.7 - 0.7) | 0.972 | -0.01 (-0.04 - 0.02) | 0.641 |
| rs3804100 | a | 0.1 (-0.6 - 0.7) | 0.915 | 0.001 (-0.03 - 0.03) | 0.967 |
| rs1816702 | a | -0.3 (-0.8 - 0.3) | 0.332 | 0.01 (-0.01 - 0.04) | 0.313 |
|  | b | -1.2 (-2.5 - 0.2) | 0.081 | 0.02 (-0.04 - 0.1) | 0.481 |
| rs11938228 | a | 0.5 (0.1 - 1.0) | **0.041** | -0.03 (-0.05 - -0.01) | **0.021** |
|  | b | 0.1 (-0.7 - 0.9) | 0.777 | 0.01 (-0.03 - 0.04) | 0.842 |
| rs7656411 | a | 0.1 (-0.5 - 0.5) | 0.921 | -0.02 (-0.04 - 0.01) | 0.098 |
|  | b | -0.3 (-1.3 - 0.6) | 0.507 | -0.002 (-0.04 - 0.04) | 0.944 |
| rs5743704 | a | -0.7 (-1.5 - 0.1) | 0.085 | 0.04 (-0.0001 - 0.07) | 0.051 |
| rs5743708 | a | -0.2 (-0.9 - 0.5) | 0.540 | -0.01 (-0.04 - 0.02) | 0.567 |
| rs4696480 | a | 0.4 (-0.2 - 0.9) | 0.177 | -0.01 (-0.03 - 0.02) | 0.518 |
|  | b | 0.2 (-0.5 - 0.8) | 0.631 | 0.001 (-0.03 - 0.03) | 0.985 |

Baseline analysis are adjusted for age, gender, pack-year, current smoking; Change analysis are adjusted for neutrophils at baseline, age at baseline, gender, current smoking at baseline, treatment, the period when there is a change in treatment and its interaction with treatment and their interaction with time; a= heterozygotes vs. wild-type; b= homozygote variant vs. wild-type.
