# Supplementary material for: Toll-Like Receptor (TLR2 and TLR4) Polymorphisms and Chronic Obstructive Pulmonary Disease
Source: PLoS One. 2012 Aug 28;7(8):e43124. doi: 10.1371/journal.pone.0043124 (PMC3429472; doi:10.1371/journal.pone.0043124)
Supplement: Table S4 — TLR2 SNPs and macrophages in induced sputum. Baseline analysis are adjusted for age, gender, pack-year, current smoking; Change analysis are adjusted for macrophages at baseline, age at baseline, gender, current smoking at baseline, treatment, the period when there is a change in treatment and its interaction with treatment and their interaction with time; a = heterozygotes vs. wild-type; b = homozygote variant vs. wild-type. (DOC) [file pone.0043124.s005.doc]

**Table S4: *TLR2* SNPs and macrophages in induced sputum**

| **SNP** |  | | **(ln)macrophages** | **p** | **(ln)macrophages** | **p** |
| --- | --- | --- | --- | --- | --- | --- |
|  |  | | **baseline** |  | **change** |  |
|  |  | | **B (95%CI)** |  | **E (95%CI)** |  |
| rs1898830 | | a | 0.4 (-0.1 - 0.8) | 0.083 | -0.002 (-0.03 - 0.02) | 0.907 |
|  | | b | -0.5 (-1.3 - 0.3) | 0.236 | 0.04 (-0.01 - 0.08) | 0.087 |
| rs3804099 | | a | 0.3 (-0.2 - 0.8) | 0.178 | -0.04 (-0.06 - - 0.01) | **0.006** |
|  | | b | 0.4 (-0.6 - 0.6) | 0.886 | -0.02 (-0.05 - 0.01) | 0.226 |
| rs3804100 | | a | -0.1 (-0.7 - 0.5) | 0.811 | -0.03 (-0.06 - 0.01) | 0.083 |
| rs1816702 | | a | -0.3 (-0.7 - 0.2) | 0.289 | 0.02 (-0.01 - 0.04) | 0.212 |
|  | | b | -0.5 (-1.6 - 0.8) | 0.464 | 0.03 (-0.03 - 0.08) | 0.289 |
| rs11938228 | | a | 0.5 (0.1 - 0.9) | **0.027** | -0.01 (-0.03 - 0.02) | 0.546 |
|  | | b | 0.1 (-0.6 - 0.8) | 0.748 | 0.02 (-0.02 - 0.05) | 0.408 |
| rs7656411 | | a | 0.1 (-0.3 - 0.6) | 0.577 | -0.02 (-0.05 - 0.01) | 0.066 |
|  | | b | 0.1 (-0.9 - 0.7) | 0.793 | -0.03 (-0.07 - 0.02) | 0.267 |
| rs5743704 | | a | -0.8 (-1.5 - -0.1) | **0.027** | 0.02 (-0.03 - 0.06) | 0.453 |
| rs5743708 | | a | -0.2 (-0.9 - 0.4) | 0.496 | 0.01 (-0.02 - 0.04) | 0.527 |
| rs4696480 | | a | 0.1 (-0.4 - 0.6) | 0.604 | -0.01 (-0.04 - 0.01) | 0.302 |
|  | | b | -0.1 (-0.6 - 0.5) | 0.802 | -0.03 (-0.05 - 0.01) | 0.096 |

Baseline analysis are adjusted for age, gender, pack-year, current smoking; Change analysis are adjusted for macrophages at baseline, age at baseline, gender, current smoking at baseline, treatment, the period when there is a change in treatment and its interaction with treatment and their interaction with time; a= heterozygotes vs. wild-type; b= homozygote variant vs. wild-type.
