# Supplementary material for: Toll-Like Receptor (TLR2 and TLR4) Polymorphisms and Chronic Obstructive Pulmonary Disease
Source: PLoS One. 2012 Aug 28;7(8):e43124. doi: 10.1371/journal.pone.0043124 (PMC3429472; doi:10.1371/journal.pone.0043124)
Supplement: Table S5 — TLR2 SNPs and eosinophils in induced sputum. Baseline analysis are adjusted for age, gender, pack-year, current smoking; Change analysis are adjusted for eosinophils at baseline, age at baseline, gender, current smoking at baseline, treatment, the period when there is a change in treatment and its interaction with treatment and their interaction with time; a = heterozygotes vs. wild-type; b = homozygote variant vs. wild-type. (DOC) [file pone.0043124.s006.doc]

**Table S5: *TLR2* SNPs and eosinophils in induced sputum**

| **SNP** |  | | **(ln)eosinophils** | **p** | **(ln)eosinophils** | **p** |
| --- | --- | --- | --- | --- | --- | --- |
|  |  | | **baseline B** |  | **change** |  |
|  |  | | **(95%CI)** |  | **E (95%CI)** |  |
| rs1898830 | | a | 0.5 (-0.2 - 1.1) | 0.134 | -0.02 (-0.05 - 0.02) | 0.273 |
|  | | b | 0.5 (-0.7 - 1.7) | 0.399 | -0.03 (-0.09 - 0.04) | 0.433 |
| rs3804099 | | a | -0.5 (-1.2 - 0.2) | 0.167 | -0.01 (-0.04 - 0.03) | 0.721 |
|  | | b | -1.1 (-1.91 - -0.2) | **0.015** | -0.01 (-0.04 - 0.05) | 0.790 |
| rs3804100 | | c | -0.4 (-1.3 - 0.5) | 0.604 | 0.01 (-0.04 - 0.06) | 0.664 |
| rs1816702 | | a | -0.1 (-0.8 - 0.6) | 0.735 | 0.03 (-0.01 - 0.06) | 0.118 |
|  | | b | -1.1 (-2.9 - 0.8) | 0.251 | 0.01 (-0.07 - 0.09) | 0.812 |
| rs11938228 | | a | 0.7 (0.03 - 1.3) | **0.043** | -0.02 (-0.06 - 0.01) | 0.179 |
|  | | b | 0.9 (-0.1 - 1.9) | 0.071 | -0.04 (-0.09 - 0.02) | 0.199 |
| rs7656411 | | a | -0.3 (-0.9 - 0.4) | 0.483 | -0.002 (-0.04 - 0.03) | 0.922 |
|  | | b | -1.6 (-2.9 - 0.4) | **0.012** | -0.01 (-0.06 - 0.07) | 0.820 |
| rs5743704 | | a | -1.0 (-2.1 - 0.1) | 0.070 | 0.04 (-0.02 - 0.09) | 0.141 |
| rs5743708 | | a | 0.1 (-0.9 - 1.02) | 0.879 | -0.02 (-0.06 - 0.03) | 0.421 |
| rs4696480 | | a | -0.1 (-0.8 - 0.7) | 0.872 | 0.03 (-0.01 - 0.06) | 0.168 |
|  | | b | -0.5 (-1.4 - 0.3) | 0.210 | 0.01 (-0.04 - 0.05) | 0.745 |

Baseline analysis are adjusted for age, gender, pack-year, current smoking; Change analysis are adjusted for eosinophils at baseline, age at baseline, gender, current smoking at baseline, treatment, the period when there is a change in treatment and its interaction with treatment and their interaction with time; a= heterozygotes vs. wild-type; b= homozygote variant vs. wild-type.
