# Supplementary material for: Toll-Like Receptor (TLR2 and TLR4) Polymorphisms and Chronic Obstructive Pulmonary Disease
Source: PLoS One. 2012 Aug 28;7(8):e43124. doi: 10.1371/journal.pone.0043124 (PMC3429472; doi:10.1371/journal.pone.0043124)
Supplement: Table S6 — TLR2 SNPs and epithelial cells in induced sputum. Baseline analysis are adjusted for age, gender, pack-year, current smoking; Change analysis are adjusted for epithelial cells at baseline, age at baseline, gender, current smoking at baseline, treatment, the period when there is a change in treatment and its interaction with treatment and their interaction with time; a = heterozygotes vs. wild-type; b = homozygote variant vs. wild-type. (DOC) [file pone.0043124.s007.doc]

**Table S6: *TLR2* SNPs and epithelial cells in induced sputum**

| **SNP** |  | **(ln) epithelial cells** | **p** | **(ln) epithelial cells** | **p** |
| --- | --- | --- | --- | --- | --- |
|  |  | **baseline B (95%CI)** |  | **change E (95%CI)** |  |
| rs1898830 | a | -0.3 (-0.9 - 0.3) | 0.313 | 0.01 (-0.03 - 0.04) | 0.632 |
|  | b | -0.4 (-1.5 - 0.8) | 0.523 | -0.01 (-0.07 - 0.06) | 0.837 |
| rs3804099 | a | 0.4 (-0.3 - 1.02) | 0.249 | 0.01 (-0.03 - 0.05) | 0.749 |
|  | b | 1.02 (0.2 - 1.8) | **0.013** | 0.01 (-0.04 - 0.06) | 0.622 |
| rs3804100 | a | 0.3 (-0.5 - 1.1) | 0.462 | -0.01 (-0.06 - 0.04) | 0.689 |
| rs1816702 | a | 0.3 (-0.4 - 0.9) | 0.441 | 0.03 (-0.02 - 0.06) | 0.222 |
|  | b | -0.5 (-2.2 - 1.2) | 0.536 | -0.01 (-0.09 - 0.07) | 0.814 |
| rs11938228 | a | -0.2 (-0.8 - 0.5) | 0.599 | 0.02 (-0.02 - 0.05) | 0.342 |
|  | b | -0.2 (-1.1 - 0.8) | 0.727 | -0.01 (-0.07 - 0.04) | 0.671 |
| rs7656411 | a | 0.2 (-0.4 - 0.8) | 0.559 | 0.01 (-0.03 - 0.04) | 0.841 |
|  | b | 0.5 (-0.7 - 1.6) | 0.455 | -0.02 (-0.09 - 0.05) | 0.587 |
| rs5743704 | a | -0.5 (-1.6 - 0.5) | 0.298 | -0.01 (-0.07 - 0.06) | 0.911 |
| rs5743708 | a | -0.4 (-1.2 - 0.5) | 0.376 | 0.02 (-0.04 - 0.06) | 0.583 |
| rs4696480 | a | 0.1 (-0.6 - 0.7) | 0.900 | 0.03 (-0.02 - 0.06) | 0.209 |
|  | b | 0.1 (-0.6 - 0.9) | 0.751 | -0.01 (-0.05 - 0.03) | 0.642 |

Baseline analysis are adjusted for age, gender, pack-year, current smoking; Change analysis are adjusted for epithelial cells at baseline, age at baseline, gender, current smoking at baseline, treatment, the period when there is a change in treatment and its interaction with treatment and their interaction with time; a= heterozygotes vs. wild-type; b= homozygote variant vs. wild-type.
