# Supplementary material for: Toll-Like Receptor (TLR2 and TLR4) Polymorphisms and Chronic Obstructive Pulmonary Disease
Source: PLoS One. 2012 Aug 28;7(8):e43124. doi: 10.1371/journal.pone.0043124 (PMC3429472; doi:10.1371/journal.pone.0043124)
Supplement: Table S7 — TLR4 SNPs and neutrophils in induced sputum. Baseline analysis are adjusted for age, gender, pack-year, current smoking; Change analysis are adjusted for neutrophils at baseline, age at baseline, gender, current smoking at baseline, treatment, the period when there is a change in treatment and its interaction with treatment and their interaction with time; a = heterozygotes vs. wild-type; b = homozygote variant vs. wild-type. (DOC) [file pone.0043124.s008.doc]

**Table S7: *TLR4* SNPs and neutrophils in induced sputum**

| **SNP** |  | **(ln) neutrophils** | **p** | **(ln) neutrophils** | **p** |
| --- | --- | --- | --- | --- | --- |
|  |  | **baseline** |  | **change** |  |
|  |  | **B (95%CI)** |  | **E (95%CI)** |  |
| rs2770150 | a | -0.5 (-0.9 - -0.03) | **0.039** | -0.03 (-0.05 - -0.01) | **0.022** |
|  | b | 0.1 (-0.9 - 1.1) | 0.806 | -0.02 (-0.08 - 0.04) | 0.482 |
| rs2737190 | a | -0.3 (-0.8 - 0.02) | 0.237 | -0.01 (-0.03 - 0.02) | 0.784 |
|  | b | -0.8 (-1.6 - 0.02) | 0.058 | 0.01 (-0.03 - 0.05) | 0.571 |
| rs10759932 | a | -0.4 (-0.9 - 0.2) | 0.176 | -0.01 (-0.03 - 0.02) | 0.513 |
|  | b | -1.9 (-3.20 - -0.6) | **0.006** | 0.02 (-0.04 - 0.08) | 0.524 |
| rs1927911 | a | 0.01 (-0.5 - 0.5) | 0.969 | 0.001 (-0.02 - 0.02) | 0.980 |
|  | b | -0.5 (-1.4 - 0.5) | 0.336 | 0.02 (-0.03 - 0.06) | 0.523 |
| rs4986790 | a | -0.4 (-1.1 - 0.4) | 0.338 | -0.03 (-0.06 - 0.01) | 0.098 |
| rs11536889 | a | 0.1 (-0.4 - 0.6) | 0.623 | -0.01 (-0.03 - 0.01) | 0.386 |
|  | b | 1.3 (-0.1 - 2.6) | 0.061 | 0.05 (-0.01 - 0.10) | 0.098 |
| rs7856729 | a | -0.1 (-0.6 - 0.5) | 0.879 | 0.01 (-0.02 - 0.04) | 0.552 |
|  | b | 0.5 (-1.1 - 2.1) | 0.523 | 0.01 (-0.06 - 0.07) | 0.858 |
| rs7846989 | a | -0.3 (-0.9 - 0.4) | 0.463 | -0.03 (-0.05 - 0.01) | 0.087 |
|  | b | -0.8 (-3.1 - 1.6) | 0.504 | -0.08 (-0.17 - 0.01) | 0.066 |
| rs7037117 | a | 0.2 (-0.8 - 1.1) | 0.750 | -0.02 (-0.07 - 0.03) | 0.427 |
|  | b | 0.3 (-1.5 - 1.9) | 0.763 | -0.09 (-0.16 - -0.01) | **0.025** |
| rs10983755 | a | -0.7 (-1.7 - 0.4) | 0.188 | 0.02 (-0.03 - 0.07) | 0.427 |
|  | b | -0.9 (-3.1 - 1.4) | 0.456 | -0.08 (-0.17 - 0.02) | 0.097 |
| rs12377632 | a | 0.5 (0.01 - 1.0) | **0.045** | 0.002 (-0.03 - 0.03) | 0.988 |
|  | b | 1.0 (0.4 - 1.7) | **0.003** | 0.04 (0.01 - 0.07) | **0.023** |
| rs11536857 | a | -0.7 (-1.6 - 0.2) | 0.120 | -0.06 (-0.10 - -0.01) | **0.013** |
|  | b | -0.6 (-1.7 - 0.5) | 0.258 | 0.02 (-0.03 - 0.06) | 0.475 |
| rs11536869 | a | -0.5 (-1.9 - 0.8) | 0.430 | -0.02 (-0.03 - 0.07) | 0.470 |
| rs913930 | a | -0.4 (-0.9 - 1.1) | 0.128 | -0.01 (-0.03 - 0.01) | 0.426 |
|  | b | -0.8 (-1.6 - 0.1) | 0.074 | -0.01 (-0.06 - 0.04) | 0.665 |
| rs11536897 | a | 0.2 (-0.7 - 1.1) | 0.657 | -0.01 (-0.05 - 0.03) | 0.676 |
| rs10759931 | a | 0.4 (-0.2 - 0.9) | 0.187 | 7.5x10^-5^ (-0.03-0.03) | 0.995 |
|  | b | 0.9 (0.2 - 1.6) | **0.014** | 0.05 (0.02 - 0.08) | **0.004** |
| rs11536878 | a | 0.5 (-0.2 - 1.1) | 0.127 | 0.001 (-0.03 - 0.03) | 0.961 |
|  | b | -0.1 (-1.4 - 1.2) | 0.880 | 0.08 (-0.01 - 0.16) | 0.089 |

Baseline analysis are adjusted for age, gender, pack-year, current smoking; Change analysis are adjusted for neutrophils at baseline, age at baseline, gender, current smoking at baseline, treatment, the period when there is a change in treatment and its interaction with treatment and their interaction with time; a= heterozygotes vs. wild-type; b= homozygote variant vs. wild-type.
