# Supplementary material for: Toll-Like Receptor (TLR2 and TLR4) Polymorphisms and Chronic Obstructive Pulmonary Disease
Source: PLoS One. 2012 Aug 28;7(8):e43124. doi: 10.1371/journal.pone.0043124 (PMC3429472; doi:10.1371/journal.pone.0043124)
Supplement: Table S8 — TLR4 SNPs and macrophages in induced sputum. Baseline analysis are adjusted for age, gender, pack-year, current smoking; Change analysis are adjusted for macrophages at baseline, age at baseline, gender, current smoking at baseline, treatment, the period when there is a change in treatment and its interaction with treatment and their interaction with time; a = heterozygotes vs. wild-type; b = homozygote variant vs. wild-type. (DOC) [file pone.0043124.s009.doc]

**Table S8: *TLR4* SNPs and macrophages in induced sputum**

| **SNP** |  | **(ln)macrophages** | **p** | **(ln)macrophages** | **p** |
| --- | --- | --- | --- | --- | --- |
|  |  | **baseline** |  | **change** |  |
|  |  | **B (95%CI)** |  | **E (95%CI)** |  |
| rs2770150 | a | -0.3 (-0.7 - 0.2) | 0.234 | -0.02 (-0.04- -0.001) | **0.042** |
|  | b | 0.02 (-0.9 - 0.9) | 0.962 | -0.04 (-0.10 - 0.03) | 0.273 |
| rs2737190 | a | -0.3 (-0.7 - 0.2) | 0.283 | -0.01 (-0.03 - 0.02) | 0.623 |
|  | b | -0.6 (-1.4 - 0.1) | 0.096 | -0.001 (-0.04 - 0.04) | 0.970 |
| rs10759932 | a | -0.4 (-0.9 - 0.2) | 0.184 | -6.0x10^-5^ (-0.03 -0.03) | 0.996 |
|  | b | -1.2 (-2.4 - 0.1) | 0.058 | -0.01 (-0.07 - 0.05) | 0.797 |
| rs1927911 | a | -0.2 (-0.6 - 0.3) | 0.369 | 0.002 (-0.02 - 0.03) | 0.863 |
|  | b | -0.7 (-1.5 - 0.2) | 0.118 | 0.01 (-0.03 - 0.06) | 0.608 |
| rs4986790 | a | 0.1 (-0.6 - 0.7) | 0.825 | -0.04 (-0.07 - -0.003) | **0.034** |
| rs11536889 | a | 0.5 (0.02 - 0.9) | **0.043** | -0.02 (-0.04 - 0.01) | 0.206 |
|  | b | 1.2 (0.1 - 2.4) | **0.036** | 0.04 (-0.02 - 0.09) | 0.232 |
| rs7856729 | a | -0.2 (-0.7 - 0.3) | 0.411 | 0.001 (-0.03 - 0.03) | 0.941 |
|  | b | 0.3 (-1.1 - 1.8) | 0.653 | 0.04 (-0.03 - 0.10) | 0.300 |
| rs7846989 | a | 0.1 (-0.6 - 0.6) | 0.916 | -0.03 (-0.05 - 0.01) | 0.090 |
|  | b | -0.2 (-2.3 - 1.9) | 0.864 | -0.08 (-0.17 - 0.02) | 0.094 |
| rs7037117 | a | -0.2 (-1.0 - 0.6) | 0.574 | 0.01 (-0.04 - 0.05) | 0.783 |
|  | b | 0.5 (-1.0 - 1.9) | 0.535 | -0.05 (-0.12 - 0.02) | 0.164 |
| rs10983755 | a | -0.4 (-1.3 - 0.6) | 0.428 | 0.02 (-0.03 - 0.08) | 0.373 |
|  | b | -0.2 (-2.3 - 1.9) | 0.846 | -0.07 (-0.16 - 0.03) | 0.138 |
| rs12377632 | a | 0.2 (-0.3 - 0.7) | 0.354 | 0.01 (-0.02 - 0.03) | 0.667 |
|  | b | 0.9 (0.2 - 1.5) | **0.008** | 0.06 (0.02 - 0.09) | **0.002** |
| rs11536857 | a | -0.5 (-1.3 - 0.4) | 0.259 | -0.07 (-0.11 - -0.03) | **0.001** |
|  | b | 0.02 (-0.9 - 1.0) | 0.964 | 0.01 (-0.04 - 0.05) | 0.737 |
| rs11536869 | a | -0.7 (-1.9 - 0.5) | 0.236 | 0.04 (-0.02 - 0.10) | 0.153 |
| rs913930 | a | -0.5 (-0.9 - -0.1) | **0.037** | -0.03 (-0.05 - -0.01) | **0.031** |
|  | b | -0.4 (-1.2 - 0.3) | 0.269 | -0.01 (-0.05 - 0.04) | 0.692 |
| rs11536897 | c | 0.3 (-0.6 - 1.1) | 0.537 | -0.03 (-0.07 - 0.02) | 0.227 |
| rs10759931 | a | 0.2 (-0.4 - 0.6) | 0.567 | 0.01 (-0.02 - 0.03) | 0.700 |
|  | b | 0.8 (0.1 - 1.4) | **0.019** | 0.07 (0.04 - 0.10) | **5.9x10^-5^** |
| rs11536878 | a | 0.2 (-0.4 - 0.8) | 0.441 | -0.001 (-0.03 - 0.03) | 0.982 |
|  | b | -0.8 (-1.9 - 0.3) | 0.156 | 0.09 (-0.002 - 0.18) | 0.056 |

Baseline analysis are adjusted for age, gender, pack-year, current smoking; Change analysis are adjusted for macrophages at baseline, age at baseline, gender, current smoking at baseline, treatment, the period when there is a change in treatment and its interaction with treatment and their interaction with time; a= heterozygotes vs. wild-type; b= homozygote variant vs. wild-type.
