# Supplementary material for: Toll-Like Receptor (TLR2 and TLR4) Polymorphisms and Chronic Obstructive Pulmonary Disease
Source: PLoS One. 2012 Aug 28;7(8):e43124. doi: 10.1371/journal.pone.0043124 (PMC3429472; doi:10.1371/journal.pone.0043124)
Supplement: Table S9 — TLR4 SNPs and eosinophils in induced sputum. Baseline analysis are adjusted for age, gender, pack-year, current smoking; Change analysis are adjusted for eosinophils at baseline, age at baseline, gender, current smoking at baseline, treatment, the period when there is a change in treatment and its interaction with treatment and their interaction with time; a = heterozygotes vs. wild-type; b = homozygote variant vs. wild-type. (DOC) [file pone.0043124.s010.doc]

**Table S9: *TLR4* SNPs and eosinophils in induced sputum**

| **SNP** |  | **(ln)eosinophils** | **p** | **(ln)eosinophils** | **p** |
| --- | --- | --- | --- | --- | --- |
|  |  | **baseline B (95%CI)** |  | **change E (95%CI)** |  |
| rs2770150 | a | -0.5 (-1.1 - 0.2) | 0.129 | -0.05 (-0.08 - -0.02) | **0.003** |
|  | b | -1.0 (-2.3 - 0.3) | 0.112 | -0.07 (-0.16 - 0.02) | 0.097 |
| rs2737190 | a | -0.1 (-0.7 - 0.6) | 0.828 | -0.01 (-0.04 - 0.03) | 0.653 |
|  | b | -0.6 (-1.7 - 0.6) | 0.321 | 0.05 (-0.01 - 0.11) | 0.084 |
| rs10759932 | a | -0.2 (-1.0 - 0.6) | 0.570 | -0.02 (-0.06 - 0.02) | 0.219 |
|  | b | -0.6 (-2.5 - 1.3) | 0.532 | 0.09 (0.01 - 0.17) | **0.035** |
| rs1927911 | a | -0.3 (-0.9 - 0.4) | 0.436 | -0.01 (-0.04 - 0.03) | 0.841 |
|  | b | -0.1 (-1.3 - 1.2) | 0.954 | 0.09 (0.02 - 0.15) | **0.013** |
| rs4986790 | a | 0.1 (-0.9 - 1.0) | 0.940 | -0.04 (-0.08 - 0.02) | 0.158 |
| rs11536889 | a | 0.5 (-1.02 - 1.2) | 0.143 | 0.01 (-0.03 - 0.04) | 0.852 |
|  | b | 0.1 (-1.7 - 1.9) | 0.919 | 0.10 (0.02 - 0.18) | **0.012** |
| rs7856729 | a | -0.2 (-1.0 - 0.5) | 0.546 | 0.03 (-0.01 - 0.07) | 0.149 |
|  | b | 1.3 (-0.9 - 3.4) | 0.236 | 0.08 (-0.05 - 0.19) | 0.220 |
| rs7846989 | a | 0.1 (-0.8 - 1.0) | 0.825 | -0.03 (-0.07 - 0.02) | 0.204 |
|  | b | -0.9 (-4.0 - 2.3) | 0.573 | -0.03 (-0.17 - 0.11) | 0.719 |
| rs7037117 | a | 0.2 (-1.2 - 1.7) | 0.756 | -0.05 (-0.12 - 0.03) | 0.196 |
|  | b | 0.8 (-1.9 - 3.4) | 0.562 | -0.07 (-0.22 - 0.08) | 0.339 |
| rs10983755 | a | -0.1 (-1.5 - 1.3) | 0.876 | 0.03 (-0.05 - 0.10) | 0.448 |
|  | b | -1.0 (-4.1 – 2.1) | 0.536 | -0.02 (-0.15 - 0.11) | 0.760 |
| rs12377632 | a | 1.2 (0.5 - 1.9) | **0.001** | -0.01 (-0.04 - 0.03) | 0.815 |
|  | b | 1.2 (0.3 - 2.1) | **0.011** | 0.07 (0.02 - 0.11) | **0.007** |
| rs11536857 | a | -1.4 (-2.6 - -0.2) | **0.020** | -0.7 (-0.13 - 0.001) | 0.053 |
|  | b | 0.3 (-1.2 - 1.7) | 0.738 | -0.01 (-0.08 - 0.05) | 0.679 |
| rs11536869 | a | -0.2 (-2.0 - 1.6) | 0.831 | 0.01 (-0.08 - 0.08) | 0.935 |
| rs913930 | a | -0.5 (-1.1 - 0.1) | 0.105 | -0.04 (-0.07 - -0.01) | **0.023** |
|  | b | -1.7 (-2.8 - -0.6) | **0.003** | -0.07 (-0.13 - -0.01) | **0.037** |
| rs11536897 | a | 0.5 (-0.7 - 1.7) | 0.427 | 0.02 (-0.05 - 0.08) | 0.578 |
| rs10759931 | a | 0.9 (0.2 - 1.7) | **0.013** | 0.002 (-0.04 - 0.04) | 0.903 |
|  | b | 0.9 (-0.1 - 1.8) | 0.079 | 0.06 (0.02 - 0.11) | **0.006** |
| rs11536878 | a | -1.3x10^-5^ (-0.9- 0.9) | 1.000 | 0.03 (-0.02 - 0.08) | 0.168 |
|  | b | 0.9 (-0.9 - 2.7) | 0.308 | 0.08 (-0.05 - 0.21) | 0.231 |

Baseline analysis are adjusted for age, gender, pack-year, current smoking; Change analysis are adjusted for eosinophils at baseline, age at baseline, gender, current smoking at baseline, treatment, the period when there is a change in treatment and its interaction with treatment and their interaction with time; a= heterozygotes vs. wild-type; b= homozygote variant vs. wild-type.
