# Supplementary material for: Toll-Like Receptor (TLR2 and TLR4) Polymorphisms and Chronic Obstructive Pulmonary Disease
Source: PLoS One. 2012 Aug 28;7(8):e43124. doi: 10.1371/journal.pone.0043124 (PMC3429472; doi:10.1371/journal.pone.0043124)
Supplement: Table S10 — TLR4 SNPs and epithelial cells in induced sputum. Baseline analysis are adjusted for age, gender, pack-year, current smoking; Change analysis are adjusted for epithelial cells at baseline, age at baseline, gender, current smoking at baseline, treatment, the period when there is a change in treatment and its interaction with treatment and their interaction with time; a = heterozygotes vs. wild-type;b = homozygote variant vs. wild-type. (DOC) [file pone.0043124.s011.doc]

**Table S10: *TLR4* SNPs and epithelial cells in induced sputum**

| **SNP** |  | **(ln) epithelial cells** | **p** | **(ln) epithelial cells** | **p** |
| --- | --- | --- | --- | --- | --- |
|  |  | **baseline B (95%CI)** |  | **change E (95%CI)** |  |
| rs2770150 | a | 0.1 (-0.5 - 0.7) | 0.772 | -0.04 (-0.07- -0.002) | **0.035** |
|  | b | -0.6 (-1.9 - 0.6) | 0.310 | -0.09 (-0.19 - 0.01) | 0.060 |
| rs2737190 | a | -0.6 (-1.2 - 0.1) | 0.068 | 0.02 (-0.02 - 0.05) | 0.375 |
|  | b | -0.2 (-1.3 - 0.8) | 0.660 | 0.04 (-0.02 - 0.10) | 0.192 |
| rs10759932 | a | -0.6 (-1.3 - 0.2) | 0.108 | 0.02 (-0.02 - 0.06) | 0.279 |
|  | b | 0.1 (-1.7 - 1.7) | 0.964 | 0.04 (-0.05 - 0.13) | 0.373 |
| rs1927911 | a | -0.7 (-1.3 - 0.1) | **0.022** | 0.04 (0.01 - 0.08) | **0.030** |
|  | b | -0.3 (-1.4 - 0.8) | 0.591 | 0.04 (-0.03 - 0.11) | 0.235 |
| rs4986790 | a | 0.5 (-0.5 - 1.4) | 0.299 | -0.05 (-0.11 - 0.01) | 0.072 |
| rs11536889 | a | 0.3 (-0.4 - 0.9) | 0.443 | -0.03 (-0.06 - 0.01) | 0.166 |
|  | b | 0.6 (-1.1 - 2.2) | 0.488 | 0.02 (-0.07 - 0.10) | 0.692 |
| rs7856729 | a | -0.5 (-1.2 - 0.2) | 0.147 | 0.04 (0.001 - 0.09) | **0.048** |
|  | b | 01. (-1.9 - 2.1) | 0.922 | 0.01 (-0.09 -0.11) | 0.812 |
| rs7846989 | a | 0.7 (-0.1 - 1.5) | 0.081 | -0.03 (-0.07 - 0.02) | 0.252 |
|  | b | 1.3 (-1.5 - 4.1) | 0.364 | 0.03 (-0.12 - 0.17) | 0.724 |
| rs7037117 | a | -0.6 (-1.5 - 0.3) | 0.173 | 0.03 (-0.03 - 0.09) | 0.270 |
|  | b | 0.5 (-1.2 - 2.2) | 0.551 | -0.01(-0.09 - 0.09) | 0.955 |
| rs10983755 | a | -0.9 (-2.2 - 0.4) | 0.166 | -0.02 (-0.10 - 0.06) | 0.656 |
|  | b | 1.1 (-1.8 - 3.9) | 0.456 | 0.04 (-0.11 - 0.18) | 0.647 |
| rs12377632 | a | -0.02 (-0.7 - 0.7) | 0.952 | -0.01 (-0.04 - 0.03) | 0.792 |
|  | b | 0.9 (-0.1 - 1.8) | 0.059 | 0.03 (-0.02 - 0.08) | 0.278 |
| rs11536857 | a | 0.1 (-1.0 - 1.2) | 0.875 | 0.01 (-0.06 - 0.08) | 0.872 |
|  | b | 0.4 (-1.0 - 1.7) | 0.579 | -0.05 (-0.11 - 0.02) | 0.190 |
| rs11536869 | a | 0.01 (-1.7 - 1.7) | 0.991 | 0.01 (-0.08 - 0.09) | 0.916 |
| rs913930 | a | -0.4 (-1.0 - 0.3) | 0.228 | -0.02 (-0.06 - 0.01) | 0.198 |
|  | b | -0.5 (-1.6 - 0.6) | 0.358 | -0.002 (-0.07 - 0.07) | 0.967 |
| rs11536897 | a | -0.02 (-1.2 - 1.1) | 0.966 | 0.03 (-0.04 - 0.09) | 0.409 |
| rs10759931 | a | -0.1 (-0.7 - 0.6) | 0.894 | 0.002 (-0.04 - 0.04) | 0.906 |
|  | b | 0.8 (-0.1 - 1.6) | 0.084 | 0.03 (-0.02 - 0.08) | 0.257 |
| rs11536878 | a | -0.4 (-1.2 - 0.5) | 0.376 | 0.04 (-0.02 - 0.08) | 0.175 |
|  | b | -0.7 (-2.4 - 0.9) | 0.389 | 0.03 (-0.12 - 0.17) | 0.737 |

Baseline analysis are adjusted for age, gender, pack-year, current smoking; Change analysis are adjusted for epithelial cells at baseline, age at baseline, gender, current smoking at baseline, treatment, the period when there is a change in treatment and its interaction with treatment and their interaction with time; a= heterozygotes vs. wild-type;b= homozygote variant vs. wild-type.
